# Potential Anti-SARS-CoV-2 Molecular Strategies

**DOI:** 10.3390/molecules28052118

**Published:** 2023-02-24

**Authors:** Caterina Vicidomini, Giovanni N. Roviello

**Affiliations:** Institute of Biostructures and Bioimaging, Italian National Council for Research (IBB-CNR), Area di Ricerca Site and Headquarters, Via Pietro Castellino 111, 80131 Naples, Italy

## Abstract

Finding effective antiviral molecular strategies was a main concern in the scientific community when the severe acute respiratory syndrome coronavirus 2 (SARS-CoV-2) emerged at the end of 2019 as an easily transmissible and potentially deadly β-coronavirus able to cause the coronavirus disease 19 (COVID-19), which famously led to one of the most worrying pandemics in recent times. Other members of this zoonotic pathogenic family were already known before 2019, but apart from the SARS-CoV, which was responsible of severe acute respiratory syndrome (SARS) pandemic in 2002/2003, and Middle East respiratory syndrome coronavirus (MERS-CoV), whose main impact on humans is geographically restricted to Middle Eastern countries, the other human β-coronaviruses known at that time were those typically associated with common cold symptoms which had not led to the development of any specific prophylactic or therapeutic measures. Although SARS-CoV-2 and its mutations are still causing illness in our communities, COVID-19 is less deadly than before and we are returning to normality. Overall, the main lesson learnt after the past few years of pandemic is that keeping our bodies healthy and immunity defenses strong using sport, nature-inspired measures, and using functional foods are powerful weapons for preventing the more severe forms of illness caused by SARS-CoV-2 and, from a more molecular perspective, that finding drugs with mechanisms of action involving biological targets conserved within the different mutations of SARS-CoV-2—and possibly within the entire family of β-coronaviruses—gives more therapeutic opportunities in the scenario of future pandemics based on these pathogens. In this regard, the main protease (M^pro^), having no human homologues, offers a lower risk of off-target reactivity and represents a suitable therapeutic target in the search for efficacious, broad-spectrum anti-β-coronavirus drugs. Herein, we discuss on the above points and also report some molecular approaches presented in the past few years to counteract the effects of β-coronaviruses, with a special focus on SARS-CoV-2 but also MERS-CoV.

## 1. Molecules against β-Coronaviruses

It is well known that the coronavirus disease 19 (COVID-19), caused by the severe acute respiratory syndrome coronavirus 2 (SARS-CoV-2), has caused enormous sanitary and socio-economic difficulties to the entire world due to the high transmission rates of the virus, the lack of effective treatments available when it first emerged, and the rapid genetic mutations of the virus observed in recent years. Among the biomolecules crucial for the viral processes, the spike protein is a key structural protein that mediates host infection by SARS-CoV-2, with the structure of the N-terminal signal peptide of this SARS-CoV-2 protein having an effect on the synthesis and secretion of the spike, which suggests that developing drugs which target the signaling peptide of the spike could be a winning anti-COVID-19 strategy [1]. Moreover, studies conducted on the S1 subunit of this protein have revealed the inactivation abilities of ozonated water and slightly acidic electrolyzed water and ethanol, and these were proposed as effective as SARS-CoV-2 disinfectants [2]. Another relevant SARS-CoV-2 protein target is nucleoprotein, also known as protein N or nucleocapsid protein, an abundant RNA-binding protein critical for viral genome packaging and essential for the replication machinery of SARS-CoV-2 [3]. It is a potential link between viral replication and the multiple signaling pathways which lead to long-lasting COVID-19 symptoms, and new ligands of the protein N were investigated by computational and experimental studies based on dynamic light scattering and surface plasmon resonance, which led to the identification of substance P(1-7) and enkephalins as effective binders of the major sites at the C-terminus or of β-sheets at the N-terminus of the viral protein. These studies led to the conclusion that antiviral drugs that target N can contribute to reducing brain fog and stroke risk and improving COVID-19 patient well-being [3]. One of the most widely explored strategies for lowering virus transmission and disease severity has been COVID-19 vaccination. Vaccines are a powerful weapon which can be used to prevent the worrying scenarios caused by a number of pathogens [4], and have been widely employed in the form of mass vaccination during the COVID-19 pandemic. Sadly, COVID-19 vaccines are not exempt from side effects, including, in some cases, thrombotic thrombocytopenia syndrome, which led to some specific COVID-19 vaccines being discouraged regarding their use in younger individuals [5]. Remarkably, vaccination responses vary significantly depending on the host, even though correlations with protection against SARS-CoV-2 were reported [6]. However, despite vaccinations, the recurrence of SARS-CoV-2 infection was frequently observed also in vaccinated individuals, while disease can still severely affect elderly and hospitalized patients who are fragile and vulnerable, which recalls the need for new efforts to be devoted to the discovery of potential global therapeutics, utilizable in the context of the infections of new mutations of SARS-CoV-2, but possibly also of other human β-coronaviruses. COVID-19 causes widespread respiratory and non-respiratory symptoms and, regardless of the underlying comorbid conditions, early treatment of COVID-19 has the potential to positively affect the clinical course of the disease. Moreover, even after the virus has been eradicated, the previous SARS-CoV-2 interaction with host cell receptors and consequent activation of pro-inflammatory pathways may still be responsible for endothelial and epithelial damage mechanisms. In this context, anti-inflammatory treatments, such as steroidal and non-steroidal drugs, as well as cytokine inhibitors, have been suggested in the treatment of COVID-19 patients, since inflammation treatment has been identified as a crucial step in the recovery process [7]. The complications observed in COVID-19 patients are numerous, especially in cases of the more severe forms of the diseases. For example, a strong correlation between the impaired renal function and in-hospital deaths of critically ill COVID-19 patients, particularly those with comorbidities and requiring renal replacement therapy, has been supported by data from numerous intensive care units around the world [8]. The common renal disorder known as acute kidney injury, characterized by a sudden and persistent decline in renal function, is sometimes experienced by COVID-19 patients during their hospitalization, with a significant impact on their survival. Catechins were also found to be effective therapeutics in COVID-19-associated acute kidney injury thanks to their wide range of pharmacological effects, which included anti-coronavirus, antioxidant, anti-inflammatory, and renoprotective effects against kidney damage [8]. More geographically confined than SARS-CoV-2, but also much more deadly, Middle East respiratory syndrome coronavirus (MERS-CoV) is another highly pathogenic zoonotic β-coronavirus, first discovered in patients living in the Arabian Peninsula in September 2012, that has an unusually high fatality rate in humans and causes severe, frequently fatal respiratory illness [9]. Natural products were investigated as MERS drugs using approaches employing quantum mechanics calculations, pharmacophore-based virtual screening, and molecular dynamics simulations, revealing the potential of compounds such as taiwanhomoflavone B, 2,3-dihydrohinokiflavone, and sophoricoside as effective natural anti-MERS-CoV candidates [9].

## 2. Drug Repositioning

**Figure 1:**
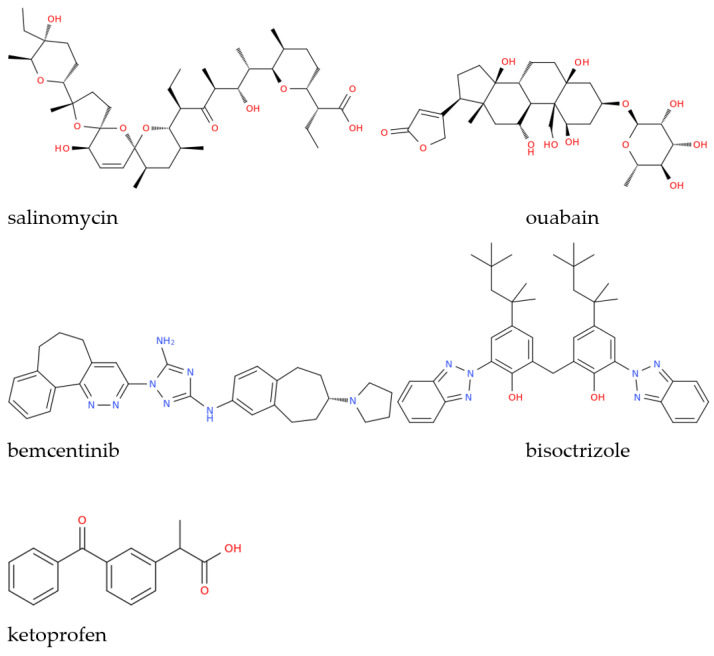
Structural representation of some of the drugs investigated as anti-COVID-19 therapeutics mentioned in this work.

Drug repositioning using previously FDA-approved drugs is a strategy typically employed when a new pathogen emerges in the attempt to treat patients who need urgent cures while waiting for more specific therapies to be developed, which clearly takes a longer time. This was especially true in the context of COVID-19, regarding which countless studies, often based on computational approaches using molecular docking and molecular dynamics simulations, were conducted on different molecular targets of SARS-CoV-2, including the papain-like protease (PL^pro^), RNA-dependent RNA polymerase (RdRp), and SARS-CoV-2 main protease (M^pro^), to name only a few. This led to the identification of salinomycin (Figure 1) from *Streptomyces albus* as a potential inhibitor of SARS-CoV-2 PL^pro^, while the vegetal toxin ouabain was proposed as a dual inhibitor of the PL^pro^ and M^pro^ enzymes [10]. By screening 171 candidates obtained from the DrugBank database (http://www.drugbank.ca/ accessed on 17 February 2023), other in silico studies identified possible organic triazole compounds such as bemcentinib, and bisoctrizole, as M^pro^ inhibitors whose pharmacokinetic characteristics were also evaluated, and their complex stability and conformation were examined using molecular dynamics simulation [11]. Non-steroidal anti-inflammatory drugs, which are frequently used to treat upper airway infections symptomatically, are of crucial importance when administered in the early stages of SARS-CoV-2 infection and, in this context, ketoprofen lysine salt is a non-steroidal anti-inflammatory drug which was suggested to offer notable benefits in early COVID-19 therapy, based on the pharmacodynamic and pharmacokinetic characteristics of this drug [7].

## 3. Nature against β-Coronaviruses

Since nature is an inexhaustible source of remedies and therapeutic scaffolds, new potential drugs to be used in the fight against COVID-19 were often searched for from natural sources, especially plants [12,13,14], using highly diverse approaches such as transcriptomics, cheminformatics, and systems pharmacology with a holistic view [15]. Natural inhibitors of SARS-CoV-2 nonstructural protein (nsp10) were screened from a database containing 310 naturally isolated metabolites through various in silico selection methods including molecular similarity assessment, molecular fingerprint, docking studies, toxicity, ADMET (absorption, distribution, metabolism, excretion, and toxicity), and density-functional theory [16]. Polyphenol-rich tea leaf extracts containing concentrated theaflavins and several virucidal catechins were tested for their ability to inactivate SARS-CoV-2 in cellular studies and the virus structural proteins and viral RNA were also examined using Western blotting and real-time RT-PCR to assess the effects of the tea leaf extracts on viral proteins and the viral genome. Remarkably, cellular infection by SARS-CoV-2 was prevented by the treatment using natural preparations, which, from a molecular point of view, caused structural changes of the S2 subunit of the spike protein and damages to the viral genome, giving clues to the potential efficacy of tea leaf extracts as virucidal agents in vitro, which could improve the existing control measures adopted against β-coronaviruses [17]. In the context of the application of traditional and herbal medicine to the therapy and prevention of infections caused by β-coronaviruses, we have suggested the anti-COVID-19 effects of cloves (*Syzygium aromaticum* L.). This is a culinary spice with a long history of use in folk medicine for a variety of disorders, being used in traditional medicine to treat respiratory ailments since ancient times. Among its molecular ingredients, different clove phytochemicals have antiviral, anti-inflammatory, antibacterial, immunostimulatory, and antithrombotic properties, all useful aspects in the context of developing an effective anti-COVID-19 therapy [18]. In conclusion, more research on COVID-19 therapies making use of synthetic and natural molecules and their derivatives is unquestionably required. In fact, the scientific efforts regarding both therapy and the prophylaxis of β-coronavirus diseases should not be discontinued even if currently COVID-19 appears to be less deadly, because SARS-CoV-2 could re-emerge with more pathogenic variants and, moreover, it is not unlikely that, with the increased rate of human–wildlife contact due to the loss of intact ecosystems and forested areas, new zoonoses caused by β-coronaviruses will continue to emerge, and humanity could have to face new pandemics in the near future.
